# Autism community priorities in diverse low-resource settings: A country-wide scoping exercise in India

**DOI:** 10.1177/13623613231154067

**Published:** 2023-03-31

**Authors:** Ipsita Dey, Sreerupa Chakrabarty, Rajanya Nandi, Rakshita Shekhar, Sakhi Singhi, Shoba Nayar, Jai Ranjan Ram, Shaneel Mukerji, Bhismadev Chakrabarti

**Affiliations:** 1 India Autism Center, India; 2 Auckland University of Technology, New Zealand; 3 Mental Health Foundation, India; 4 University of Reading, UK; 5 Ashoka University, India

**Keywords:** autism spectrum disorders, global, low- and middle-income country, stakeholder consultation

## Abstract

While studies to map the priorities of the autistic community have been undertaken in some high-income countries, there has been little or no such systematic assessment in the global south. India alone is home to an estimated >5 million autistic individuals. To address this gap in the literature, this study conducted a survey of the Indian autism community on their priorities for three areas: skills training, intervention and research. Individuals with a clinical diagnosis of autism spectrum disorders and parents/guardians of autistic individuals were invited to take part in the study. Data from 280 respondents were collected online and followed up with interviews on a subset (*n* = 40) chosen through stratified random selection. Results highlighted a unanimous prioritisation for self-help skills as the most important area in skills training, as it was considered the foundation for acquiring all other skills. Speech and language therapy was identified as the most important intervention for autistic individuals. Within research, identifying the most effective ways for the community to support autistic people was given the topmost priority. Researchers, clinicians and policymakers may use these insights to develop services and shape future research that is more in accord with the community’s needs.

**Lay abstract:**

It is vital to directly engage with the autism community in order to develop better services and drive the research agenda. While some studies in high-income countries have mapped the priorities of the autism community, there is a severe dearth of such efforts in the global south. Five million autistic individuals are estimated to live in India alone, and there has been little effort to map their priorities. Moreover, studies in high-income countries focused largely on research priorities, and not so much on skills training and interventions. Keeping these needs in mind, we conducted an online survey followed by an in-depth conversation with parents of autistic children and autistic adults drawn from across India. We found that the respondents reported self-help skills to be the most important for training, as they considered it fundamental for every other aspect of life. Speech and language therapy was considered to be the highest intervention priority for this group, highlighting the importance of social communication. Mental health counselling was also considered to be a high priority, but several parents identified it as being more relevant for themselves rather than for their children. Within research, the topmost priority was to understand ways in which the community can better support autistic people. We hope that these findings will help researchers, policymakers and service providers to be able to make well-informed decisions, develop relevant services and shape future research.

Autism spectrum disorder (ASD; henceforth ‘autism’) is a set of lifelong neurodevelopmental conditions characterised by difficulties in social communication, repetitive behaviour and a restricted range of interests (American Psychiatric Association, 2013). It often involves comorbid conditions such as social anxiety disorder, attention deficit/hyperactivity disorder and oppositional defiant disorder (Simonoff et al., 2008). Beyond mental health conditions, autistic individuals^1^ often experience a range of physical symptoms, leading to autism being increasingly considered from a systemic perspective (Bolton, 2009; Croen et al., 2015). Autism is also often associated with a reduced quality of life (Magiati et al., 2014) and a shorter life expectancy (Nordentoft et al., 2013).

The prevalence of autism is estimated to be over 1% globally (Elsabbagh et al., 2012; Spectrum, 2022), with some high-income countries reporting considerably higher estimates (Maenner et al., 2021). However, low- and middle-income countries (LMICs) face an additional challenge of under-diagnosis. While children with less severe ASD symptoms and with higher IQ are often diagnosed at later ages in high-income countries (HICs) (Mazurek et al., 2014), such children often remain undiagnosed in LMICs where only the most severe cases are noticed (Chauhan et al., 2019; Rudra et al., 2017). Additional challenges include a general lack of public awareness of autism and prevalent stigma and discrimination concerning mental health diagnosis, which prevent families and autistic individuals from seeking diagnosis (Hahler & Elsabbagh, 2015). Finally, in many LMICs, such as India, structural and practical barriers (e.g. limited government commitment and local resources, over-centralised health systems, failure to integrate mental health services into primary healthcare, inadequate professional training and limited local expertise) thwart early and effective diagnosis and interventions.

The pioneering work of Michael Rutter and others in the late 1970s established that autism has a strong genetic component. These observations arguably contributed to shaping the direction of autism research in the ensuing decades, with a primary focus on investigating its underlying biology. An inadvertent fallout of this focus has been that relatively little research effort has been directed to the direct assessment of the needs and priorities of the autism community.

In recent years, this gap has begun to be addressed primarily in HIC (Roche et al., 2021), which provides essential insights into the existing challenges faced by the autism community. This systematic review of research priorities revealed that the autism community largely prioritised applied research, designed to enhance the lives of autistic individuals over more basic biological research on the causes of autism (Roche et al., 2021). Research areas that the community considered most relevant focused on developing skills for autistic individuals and enabling them to get employed, ensuring their physical and mental well-being, as well as the identification of autism and making services available across the lifespan (e.g. Fletcher-Watson et al., 2017; Gotham et al., 2015; den Houting & Pellicano, 2019; Nicholas et al., 2017; Pellicano et al., 2014; Shattuck et al., 2018; Vasa et al., 2018).

Beyond research, it is essential to seek the community’s priorities for skills and interventions, as these priorities can index important differences between HIC and LMICs due to differences in ascertainment and available services. To this end, previous studies have attempted to map the skills and intervention priorities of the community in HIC settings (e.g. Ghanadzade et al., 2018; Pituch et al., 2011; Preece et al., 2017; Rodger et al., 2004; Whitaker, 2007). For instance, in a survey of 350 parents, Whitaker and others identified social development and social relationships as among the highest intervention priorities. In a qualitative study by Schaaf et al. (2015), 32 parents of children with autism reported daily living, play skills and social skills as their highest priorities for intervention. Similar results have been found in a study with parents of autistic children with low-moderate support needs, where social skills and emotional behaviour have been identified as the top two priorities in skills acquisition (Petrina et al., 2015). Unsurprisingly, Pituch et al. (2011) observed that parental intervention priorities were higher in areas where children showed the maximum deficits or some emerging capacities.

However, such exercises are lacking in LMIC settings, where the needs and challenges faced by the autism community can be significantly different from those encountered in high-resource settings (Gona et al., 2016; Samadi & McConkey, 2011). Considering that nearly 95% of children with developmental disabilities live in LMICs, a direct assessment of the challenges and priorities of the relevant stakeholders is paramount (Olusanya et al., 2018).

Specifically, within South Asia, we are not aware of any such previous exercise. This creates a significant knowledge gap, considering the large population of this region (>1.3 billion) is marked by high diversity in language, culture and socio-economic status (SES), and notable differences from HIC in the availability of services and access to specialists. An early report aptly noted that economic conditions and environmental variables not only determine the intervention process in terms of availability and accessibility of resources but also the extent to which a disability impairs the functioning of the individual or how an individual’s needs can be addressed (Daley, 2002). Factors such as symptom severity, geographic location, SES, race/ethnicity and culture are known to impact the age of the first assessment and subsequent intervention (Lagunju et al., 2014; Williams et al., 2014). These observations highlight the importance of studying the preferences and perceptions of needs of the autism community in LMIC settings since these can be different from those in HICs.

To address this gap in the literature, a nationwide survey in India was undertaken, using a similar model to that of the scoping exercises undertaken in other countries (e.g. Gotham et al., 2015; Wallace et al., 2013), to provide a systematic, empirical summary of the needs and challenges of the autism community. The overarching aim of this survey is to generate a set of priorities from the autism community, which can potentially inform the agenda for policymakers, service providers and researchers in India, and other South Asian countries.

## Methods

### Design

The study design was observational, and data were collected at a single time point from autistic individuals and/or their families.

### Setting

The study was conducted in two parts. The first, quantitative part of the study was conducted as an online ranking survey administered using Microsoft Forms. The data collection period was from October 2020 to April 2021. The sample was recruited through snowball sampling, relying heavily on contacting autistic individuals, parents of autistic individuals, professionals, non-governmental organisation (NGOs), and support organisations working in the field of autism and disability. The study was further spread through word-of-mouth, emails and WhatsApp messages and advertised on social media platforms to generate additional responses.

The second, qualitative part was conducted through interviews with a subset of individuals who indicated a willingness to be contacted later. This subset was chosen using stratified random sampling to ensure a wide representation of the following parameters: location, gender, age, support needs, relationship, age of diagnosis and family income. Detailed methods for the shortlisting criteria are provided in Supplementary Material 1. All participants were contacted by the researchers (I.D. and S.C.) through phone calls to schedule the interviews, except one autistic participant (who preferred text messages).

### Ethical considerations

The ethical approval for this study was granted by Ashoka University Institutional Review Board (IRB, Ref 16_20_Chakrabarti dated 22/09/2020). All participants indicated their consent prior to participation and were free to withdraw at any point without having to cite a reason. If the participants indicated their preference for a follow-up interview or wanted to receive an acknowledgement certificate for participation, they were asked to share their contact information.

### Community involvement statement

There was autistic representation within the research team.

### Participants

#### Sample

##### Selection criteria

Inclusion criteria for this study were as follows: (a) individuals with a clinical diagnosis of ASD using the *Diagnostic and Statistical Manual of Mental Disorders* (DSM) criteria, (b) parents/guardians of individuals diagnosed with ASD, (c) the diagnosis should have been made at least 6 months before filling out the survey and (d) participants needed to be residents of India. All participants needed to meet criteria (a) and/or (b), (c) and (d).

### Measures

The study used two instruments for data collection: a questionnaire and an interview.

#### Questionnaire

A questionnaire was custom-designed to address the aims of this study. The questionnaire included three questions, one for each of the following domains: (1) skills, (2) interventions and services and (3) research. Each question had several options, and respondents were asked to organise the options in rank order of priority, using the ‘drag and drop’ function on Microsoft Forms (see Supplementary Material 2 for all questions and instructions for these three questions).

The items of the questionnaire were based on those used in previous scoping exercises in other countries. Specifically, the research statements were adapted from a similar exercise undertaken in the United Kingdom (Wallace et al., 2013). Similarly, the list of potentially important skills and intervention options was adapted from a previous country-wide survey undertaken in North America (Autism Speaks, 2012) and factors identified in a similar exercise in other countries (Ghanadzade et al., 2018; Pituch et al., 2011). Each item and its culturally appropriate phrasing were discussed in multiple rounds by members of the study team with expertise in ground realities. New items were introduced if the study team found existing surveys from other countries to be inadequate (e.g. items on Ayurveda, Yoga and Meditation in the list of options for interventions). In addition, short explanatory blurbs were included next to each option within the skill and intervention sections, to facilitate comprehension (see Supplementary Materials 2).

The questionnaire was designed in English, and then translated in parallel to Hindi and Bengali (estimated total number of speakers >500 million), to ensure that the respondents were chosen from a wide sociolinguistic demographic. The translations were assessed by bilingual subject matter experts and iteratively refined. Before the questionnaire was sent to the target population, a pilot test was done on a sample (*N* = 5) purposively selected from the target population. Their feedback was incorporated into the questionnaire and instructions.

#### Interview

The interview schedule was prepared considering the specific options within each question, this study objectives and an understanding of the social-demographic context. The interview aimed to gain a deeper understanding of the reasons behind respondents’ preferences for the various alternatives. Questions such as why or what made them choose one option over another were the primary focus of the interview. Care was taken to word the questions in a manner that helped establish and protect the rapport between the interviewer and the interviewee (see Supplementary Materials 3). The interviews were conducted electronically over Zoom for all, but two participants due to restrictions imposed by the ongoing pandemic.

### Data analysis

Responses were excluded if they satisfied one or more of these criteria: (1) ⩽1 movement from the default ordering of options in all three categories in the three questions, (2) time taken to complete the survey ⩽5 min and (3) duplicate entries.

#### Quantitative

For each option, the mean rank and standard deviation were calculated. This process was conducted separately for each of the three questions on skills, interventions and research priorities. These mean ranks were used to index the hierarchy of priorities of each given option.

#### Qualitative

The interviews were recorded electronically and transcribed manually by bilingual researchers. From the detailed transcripts, ‘excerpts’ that highlighted respondents’ reasons for choosing a particular option at a specific rank were identified independently by two researchers. These excerpts were then reviewed and discussed jointly by all researchers in relation to the quantitative data until a consensus was reached. Participant excerpts that have been incorporated into the presentation of results below have been italicised.

## Results

### Participants

From 385 eligible responses, 280 were retained for further analysis. The following number of responses was excluded on the basis of the criteria mentioned earlier: (1) ⩽1 movement from the default ordering of options in all three categories in the three questions (*n* = 19), (2) time taken to complete the survey ⩽5 min (*n* = 47) and (3) duplicates (*n* = 39) (see Table 1 and Supplementary Materials 4 for detailed sample demographics). Out of these 280 respondents, there were 12 respondents who were related to two autistic individuals each. For these 12 respondents, both these autistic individuals were included in the demographic reports (Table 1). However, these respondents only contributed one set of responses on their priorities for research, skills and interventions. The majority of respondents were parents/caregivers, drawn from across different socio-economic strata. A large proportion (~73%) of the responses were for children or young people with autism (less than 18 years old).

**Table 1.** Characteristics of participants (N=280).

Variable	*n* (%)
*Age (in years)*	
<7	90 (30.82)
8–13	107 (36.64)
14–18	23 (7.87)
19–25	51 (17.46)
26–40	16 (5.47)
40+	5 (1.71)
*Gender*	
Male	221 (75.68)
Female	68 (23.28)
Non-binary	3 (1.02)
Age of diagnosis (in years)	
<7	256 (87.67)
8–13	19 (6.5)
14–18	3 (1.02)
19–25	8 (2.73)
26–40	5 (1.71)
40+	1 (0.34)
*Relationships*	
Self	18 (6.43)
Mother	146 (52.14)
Father	92 (32.86)
Grandmother	0 (0)
Grandfather	3 (1.07)
Brother	3 (1.07)
Sister	8 (2.86)
Uncle	2 (0.71)
Aunt	8 (2.86)
*Support needs*	
High	65 (22.26)
Medium	145 (49.65)
Low	82 (28.08)
*Monthly family income (in INR)*	
<50,000	92 (32.85)
50,000–100,000	89 (31.78)
100,000–200,000	49 (17.5)
>200,000	40 (14.28)
Did not respond	10 (3.57)
*Language in which the respondents filled the survey*	
English	246 (87.85)
Bengali	28 (10)
Hindi	6 (2.14)

A subset of 40 participants was interviewed (see Supplementary Materials 1 and 3).

### Skills

**Figure 1.**
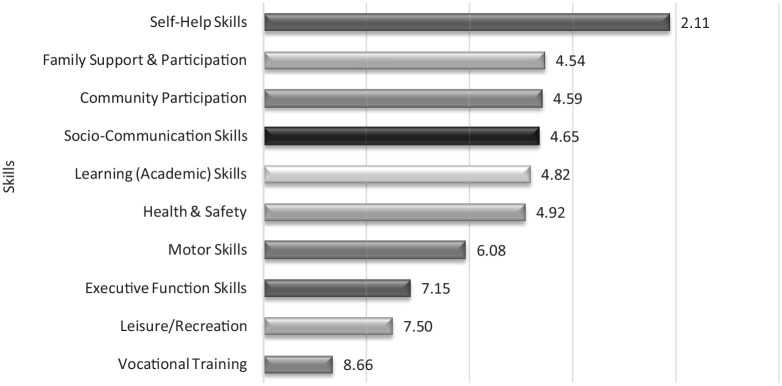
Mean ranks of the relative importance of different skill options. Options are shown in ascending rank order, with the most preferred option at the top (lowest mean rank).

The question of priorities for training skills revealed a clear prioritisation for self-help skills among the respondents (Table 2). This top priority was followed by the need for skills related to participating in family and community life. Skills related to vocation and employment received a low priority score from the current sample. Follow-up interviews revealed that the majority of the respondents felt that self-help skills constituted the foundation of all other skills. According to the respondents, when individuals can fulfil their basic needs – to eat, dress and bathe – it gives them and their caregivers a ‘sense of accomplishment’ (Figure 1).

**Table 2.** Ranks (mean, SD) given to the different skill options.

Options	Mean ranks	SD
Self-help skills	2.11	1.89
Family support and participation	4.54	2.17
Community participation	4.59	2.75
Socio-communication skills	4.65	1.95
Learning (academic) skills	4.82	2.81
Health and safety	4.92	1.98
Motor skills	6.08	2.72
Executive function skills	7.15	2.09
Leisure/recreation	7.50	1.92
Vocational training	8.66	1.95

SD: standard deviation.

To the child something like even eating your food on your own, they may be resistant in the beginning or may not even want to do it, or they may just prefer to be fed. But over a period of time, once they do these things on their own, it does give them a sense of accomplishment . . . A sense of accomplishment for the parents or the caregivers who have worked very hard to have them, see them master that skill. (Mother of a 13-year-old autistic girl)

I chose this rank because self-help and personal hygiene are most important. One should be able to do basic things and those are basic, most critical and most important- self-help or motor skills like buttoning, cutting with a scissor, catching a ball . . . That is the most basic. Without that, other skills cannot be learned. So I chose this order. (25-year-old autistic individual)

Vocational training is not the least priority. Because he’s too young now, I’m giving it the least important because a lot more skills are to be developed now. But of course, in time it will be a major priority only. (Mother of a 9-year-old autistic boy)

I don’t feel these high executive function skills and vocational skills and all . . . this is basically more apt for a high functioning child or a high functioning person. (Mother of a 26-year-old autistic man)

Learning (academic) skills have also been prioritised at a low level by the present sample. While few respondents considered it ‘secondary’ to living, others reported their children’s learning difficulties:

. . . because learning did not really help. We spent a major part of his life when he was four when we first got him diagnosed, into making him learn numbers. To date he doesn’t recognise numbers, he doesn’t recognise his name, whatever techniques, any one of them has ever used did not seem to work. And so we simply thought that this is not working for us even if he learns anything, it’s not going to help us in any way as a family. (Sibling and guardian of a 23-year-old autistic man)

Writing was not one of the things that she was enjoying . . . We kept going for it, I think from the age of 5 to around age 11 we tried making her learn alphabets or writing down, but it was something that she was not very interested in . . . but when it comes to specifically writing alphabets or writing constructively, she’s not very interested in it (Mother of a 13-year-old autistic girl)

### Interventions

**Figure 2.**
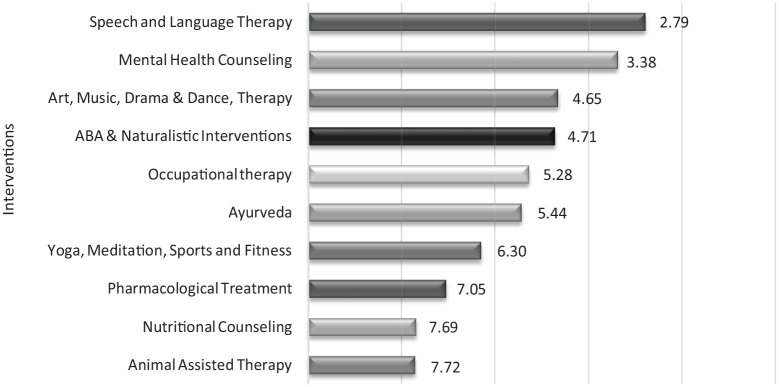
Mean ranks of the relative importance of different intervention options. Options are shown in ascending rank order, with the most preferred option at the top (lowest mean rank).

The responses to the intervention question showed a clear preference for speech and language interventions (Table 3). Follow-up interviews revealed that many parents felt that if their children were able ‘to express (their) emotions’, it could cater to meaningful communication with society at large (Figure 2).

**Table 3.** Ranks (mean, SD) given to the different intervention options.

Options	Mean ranks	SD
Speech and language therapy	2.79	1.81
Mental health counselling	3.38	2.68
Art, music, drama and dance, therapy	4.65	1.86
ABA and naturalistic interventions	4.71	2.56
Occupational therapy	5.28	2.92
Ayurveda	5.44	3.02
Yoga, meditation, sports and fitness	6.30	1.95
Pharmacological treatment	7.05	2.39
Nutritional counselling	7.69	2.37
Animal-assisted therapy	7.72	1.79

SD: standard deviation.

There are times when I would love for her to express her emotions, but I realised she just doesn’t have the literacy, vocabulary or the methodology to express her emotions in the way that we do. And as a result, I may understand her, but the world does not. (Mother of a 12-year-old autistic girl)

The second priority was mental health counselling. Interestingly, however, parents identified it as more relevant for themselves rather than their children as it would help them better understand and respond to their children’s needs:

If I take this in reference to myself, if it’s supporting the parents, then I would take it higher on the list, but if it’s for children then, if I see my son who’s not able to express himself so this does not really apply to him. (Mother of a 23-year-old autistic man)

The study also underlines respondents’ perceptions of pharmacological interventions. Many respondents expressed their preference for managing comorbid issues (e.g. sleeping disorders), without medicines; while others reported a lack of knowledge about the existence of any effective medication for autism. A specific opinion that emerged from the study was that the prioritising of pharmacological treatment would only occur if the child develops ‘any problems related to epilepsy’. Parents believed medicative treatments to ‘have a lot of side effects’. Nutritional counselling and animal-assisted therapy were ranked comparatively lower by this sample. Some interviewees also mentioned interventions/services that were not included in the list of options in the online survey. Notable among these were play therapy and special education. Overall, the interview respondents expressed their dissatisfaction with intervention services for autism, describing them as unavailable, inaccessible (only available in urban areas) and expensive. In addition, many professionals and clinicians were described as unequipped and uninformed about autism and its treatment processes:

There are not many schools where you can send them. They have neither good facilities nor great counsellors or therapists (Mother of a 13-year-old autistic girl).

I did try to find some good occupational therapy when he was young . . . I think two or three OT’s, but the condition of OT’s is pretty pathetic and I don’t know if it’s OK to say that it was not a very good experience for me, so we have not really taken any OT (Mother of a 12-year-old autistic boy).

### Research

**Figure 3.**
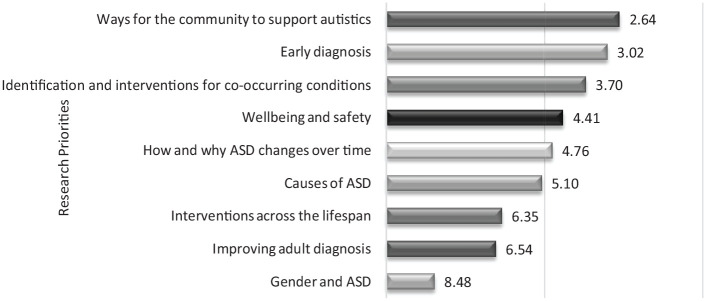
Mean ranks of the relative importance of different research statements. Options are shown in ascending rank order, with the most preferred option at the top (lowest mean rank).

In response to the question on research priorities, respondents identified ‘ways for the community to support people with ASD’ as the most important research topic (Table 4). One respondent (a mother of a 6-year-old autistic boy) commented that research on setting up ‘some kind of a social supportive framework which will take care of autistic people who require daily support’ is crucial. Early diagnosis was noted as the second most important priority by the stakeholders, as in their experience they have lost ‘valuable years’ due to ‘lack of awareness’. Interviews also elicited information about instances of bullying in childhood due to late diagnosis. Interestingly, research on gender and autism was accorded a low priority by this sample. Respondents considered ‘gender and ASD’ to be more ‘theoretical’ than capable of producing any ‘tangible outcomes’. A majority of respondents unanimously expressed their indifference to in-depth exploration of this topic, believing ‘the issues (to be) the same, whether it’s a boy or a girl’ (Mother of a 12-year-old autistic boy) (Figure 3).

**Table 4.** Ranks (mean, SD) given to the different research options.

Options	Mean ranks	SD
Ways for the community to support autistics	2.64	1.95
Early diagnosis	3.02	1.99
Identification and interventions for co-occurring conditions	3.70	1.78
Wellbeing and safety	4.41	1.69
How and why ASD changes over time	4.76	1.65
Causes of ASD	5.10	2.32
Interventions across the lifespan	6.35	2.37
Improving adult diagnosis	6.54	1.66
Gender and ASD	8.48	1.34

SD: standard deviation; ASD: autism spectrum disorder.

### Exploratory analysis

To check if monthly income influenced the ranking of the various options provided in the three questions, we conducted a median split of the sample, based on the reported monthly income. Priorities reported by the top half were compared with those reported by the bottom half, using a non-parametric comparison of means (Mann–Whitney *U* test) (Table 1 in Supplementary Material 4). On the question on skills, the only significant differences in ranking were noted for family support participation, which was given a lower priority by the higher-income group (mean rank = 5.01, standard deviation (SD) = 2.37) than the lower-income group (mean rank = 4.34, SD = 2.08). On the question of research priorities, the higher-income group gave a lower priority to early diagnosis (mean rank = 3.35, SD = 2.02) than the lower-income group (mean rank = 2.88, SD = 2.00). There were no statistically significant differences in ranking for any of the interventions.

## Discussion

Despite being home to over 5 million autistic individuals, there has been no systematic effort to map the priorities of the autism community in India. In this study, we addressed this knowledge gap by asking autistic individuals and their parents about their priorities in skills training, intervention and research through a questionnaire followed by interviews with a subset of respondents. Self-help skills were chosen as the top priority in skills, and speech and language therapy was chosen to be the top priority within interventions. Within research, the top priority was given to research into ways that the community can help to support autistic people. While some responses revealed patterns similar to those seen in high income settings, others showed interesting differences.

On the question of skills, self-help skills were considered the top priority across the board, as respondents believed such skills form the foundation for all other skills. This result is consistent with the top priority accorded to life skills training in a large survey of autistic adults in the United States (Gotham et al., 2015). The second set of skills that respondents considered most important related to participating in family and community life. Families are at the heart of Indian society, within which the individuals are embedded (Sonawat, 2001). Therefore, it is not surprising that skills that contribute to participation in family activities rank so high in the set of priorities and support. Interestingly, vocational skills ranked very low in the priorities. Since the majority of the respondents were parents with autistic children aged <13 years, one interpretation of this observation is that the respondents focused on their immediate priorities rather than long-term ones. The interviews also revealed some views on how only individuals with low support needs can get trained in vocational skills. Since the present sample consists of a majority of individuals with high and moderate support needs, it might point to why vocational skill training was rated low on the list of priorities. Finally, considering the current landscape of available services, another possibility is that the existing vocational training centres in India do not focus adequately on the specific strengths of an individual. A similar inference can be made about the relatively low priority accorded to academic/learning skills. While respondents recognised the importance of these skills, the emerging impression was that many autistic children did not enjoy the teaching style that was geared more towards their neurotypical counterparts. Taken together, these findings point to the necessity of developing training programmes and pedagogical techniques tailored to individual strengths and interests.

The question on interventions showed a strong preference for speech and language therapy. This result echoes that from a nationwide survey in North America, where parents prioritised speech therapy and occupational therapy over other interventions (Autism Speaks, 2012). This prioritisation reflects the central importance given to social communication abilities in effective day-to-day functioning. The present sample identified mental health counselling as the second most important priority. However, the interviews revealed that several respondents felt that this intervention should also target parents/caregivers. Parents of children with autism report high levels of psychological distress and are at increased risk for stress and mental health problems, even when compared to parents of children with other disabilities (Hastings, 2008). It indicates the importance of the caregivers’ psychological well-being, with sufficient coping skills for ensuring the well-being of autistic individuals. The provision of caregivers’ mental health counselling is severely limited in India, and this result points to a clear need for developing such provisions in the future. Pharmacological interventions were ranked relatively low in priority, and interviewed respondents indicated resorting to such interventions primarily for comorbid conditions such as epilepsy. Similar concerns were noted in a nationwide survey in the United Kingdom, where 60% of parents and adults with autism showed concerns about taking medications, particularly due to side effects and lack of effectiveness (Wallace et al., 2013). This result highlights the necessity for building further on non-pharmacological interventions, particularly for the core symptoms of autism. The low priority accorded to animal-assisted intervention programmes in the survey could stem from various reasons. While lack of awareness and/or availability of such programmes was a key factor for some respondents, others felt hesitant about animals being allowed inside their homes. Irrespective of the low prioritisation score, many parents mentioned noticing marked changes in their children’s behaviour when they were near animals. The eventual take-up of such interventions would need to consider traditional attitudes towards animals.

Research focusing on ways for the wider community to support autistic individuals was considered the top research priority by the current sample. This result contrasts with findings from research priority mapping exercises in HIC, which have identified the need for research into physical and mental health, skills development and service availability (Roche et al., 2021). The current result needs to be understood in an LMIC context which is marked by an absence of robust public healthcare systems and greater reliance on family and community support (Pedersen et al., 2019). A recent stakeholder consultation with young people with mental health conditions in South Asia commissioned by the Wellcome Trust revealed the critical role-played by family and community support (Huma, 2022). Early identification was a close second in the list of research priorities by the community. This result is consistent with those of the parent respondents in the United Kingdom and North American surveys (Frazier et al., 2018; Wallace et al., 2013). The gap in detecting autism early has been suggested to underpin the intervention gap for autism, particularly in LMICs (Divan et al., 2021). Since the diagnostic instruments are typically developed in HIC, their availability and applicability in LMIC are often restricted. Hence, there is a need for developing or culturally translating and validating standardised instruments considering the diverse cultural and socio-economic perspectives in LMICs (Rudra et al., 2014; Rudra et al., 2016). However, research on early diagnosis of autism manifests a divergence of views between some parents of autistic children and autistic self-advocates, specifically on prenatal identification. Parents would prefer to know of their child’s diagnosis as early as possible, including prenatally. Autistic advocates, however, were concerned that prenatal identification of autism could potentially lead to prenatal selection against autism and its eventual ‘extinction’. The relatively low priority given to research into the causes of autism is similar to results from nationwide surveys of parents and self-reported autistic people in North America (Frazier et al., 2018; Gotham et al., 2015). Research on gender and autism received a low priority in the questionnaire. However, the interviews revealed that this ranking may be driven by a potential gap in understanding. While the intended focus of this item was on the impact of biological gender on autism symptoms and services, the respondents interpreted it along the lines of gender equality – with a view that autistic males and females face very similar challenges.

An exploratory re-analysis of the data based on the reported income level of the participants revealed a largely consistent set of priorities for the respondents irrespective of income levels, except for two options. Higher priority for training in skills that help the autistic individual to take part in household tasks and family support was noted by respondents with a lower family income. Arguably, the availability of these skills can fmake a larger difference in the overall family quality of life within a lower-income setting. Interestingly, a higher priority for early diagnosis was reported by the lower-income respondents, compared to those with higher income. Receiving an early diagnosis can arguably help with the appropriate allocation of resources and interventions.

The findings of this study suggest that respondents largely prioritised the present needs of their child (or themselves, for autistic adults), similar to the findings of Pituch et al. (2011). These results underscore the necessity for helping autistic people become self-dependent through curricula that involve honing daily life skills and ensuring their overall well-being. They further suggest empowering the caregivers through relevant knowledge and support services and making the community aware of autism to provide a supportive framework. Unavailability of all necessary services under one roof, poor quality of services, and lack of availability of locally relevant services have emerged as common grievances of multiple respondents.

Despite being one of the first studies of its type in a low-resource setting, the above findings need to be considered with a few caveats. First, the study involved a web-based survey and was, therefore, only accessible to individuals with Internet access. Despite significant technological progress, Internet penetration in India is estimated to be around 50% (Basuroy, 2022) which can lead to a sampling bias. Second, snowball sampling, with its inherent advantages, may have contributed to a biased representation, with the majority of the sample being parents. Over-representation of parents is also seen in the largest survey of autism research priorities undertaken in North America (Frazier et al., 2018). The self-advocate autism community in India is still in its early stages of development due to the lack of wider recognition and diagnosis of individuals with low/medium support needs. The ongoing COVID-19 pandemic also contributed to these limitations by not allowing us to approach individuals in person. Third, the qualitative data usage is limited to the inspection of the comments made by the respondents and does not involve a formal systematic analysis or theoretical interpretation. Fourth, the survey was not appropriately designed for adults with few/no words to answer independently. This design limitation leads to the common sampling bias within autistic participants towards those whose functional language abilities are sufficient to understand and respond to the survey. Finally, we do not have any objective data on the support needs of the current sample and rely fully on the subjective reports of the respondents. Any interpretation of the results based on individual support needs must therefore be considered from within this perspective. Future studies need to address these limitations and expand upon the current effort.

## Conclusion

This study is the first systematic attempt to assess the priorities of the Indian autism community for skills, interventions and research. Self-help skills were identified as the top priority, as these were considered to be the foundation for all other skills. Speech and language interventions were considered to be of the highest priority among interventions. Identifying ways for the community to be able to support autistic individuals was considered to be the most important research area. These findings can help set the agenda for future researchers, clinicians and policymakers in the region in a manner that is closely aligned with the views of the community members. This study is expected to stimulate future large-scale scoping exercises across this region, with greater representation of self-advocates and other key stakeholders, including professionals and researchers.

## Supplemental Material

sj-docx-1-aut-10.1177_13623613231154067 – Supplemental material for Autism community priorities in diverse low-resource settings: A country-wide scoping exercise in India Click here for additional data file. Supplemental material, sj-docx-1-aut-10.1177_13623613231154067 for Autism community priorities in diverse low-resource settings: A country-wide scoping exercise in India by Ipsita Dey, Sreerupa Chakrabarty, Rajanya Nandi, Rakshita Shekhar, Sakhi Singhi, Shoba Nayar, Jai Ranjan Ram, Shaneel Mukerji and Bhismadev Chakrabarti in Autism

## Notes

1. We recognise that there is a diversity of views within the autism community on the use of person-first and identity-first language. To reflect this diversity of views, we use the terms ‘autistic person’ and ‘person with autism’ interchangeably.
