# Supplementary material for: Autism community priorities in diverse low-resource settings: A country-wide scoping exercise in India
Source: Autism. 2023 Mar 31;28(1):187–98. doi: 10.1177/13623613231154067 (PMC10771024; doi:10.1177/13623613231154067)
Supplement: sj-docx-1-aut-10.1177_13623613231154067 – Supplemental material for Autism community priorities in diverse low-resource settings: A country-wide scoping exercise in India [file sj-docx-1-aut-10.1177_13623613231154067.docx]

**Supplementary Material 1: Selection procedure for interview**

The following process was used to identify respondents for a follow-up interview.

Out of 280 individuals, 125 respondents indicated their willingness to participate in the follow-up interview. To ensure the widest possible representation, each of these demographic variables were considered separately: location, gender, age, support needs, relationship, diagnosis age, income group and total time taken to fill out the survey (as a proxy measure of engagement). For each demographic variable, all 125 data points were assigned with random numbers using the RAND () function in Microsoft Excel, and then arranged in ascending order. The first eight individuals in this order for each of these demographic variables were invited for the follow-up interview. The first 40 respondents who agreed to appear for the interview were selected for the study.

**Supplementary Material 2: Survey Questions**

**SKILLS**

Many individuals with ASD require support to learn skills. Based on your experience, please assign positions to each of these SKILLS by putting the most important on top and the least important at the bottom.

You may use the up and down arrow, or drag and drop option to adjust positions.

Community Participation: e.g., to be able to participate in and interact with people in community spaces (local restaurants, stores, parks, libraries, places of worship), and in community events (during festivals).

Self-Help Skills: e.g., to be able to serve and eat food, bathe and dress, tidy up after

oneself.

Learning (Academic) Skills: e.g., to be able to develop reading and number skills, summarize matter from a lesson, do project work, and understand technology.

Family Support & Participation: e.g., to be able to help in household jobs (like cleaning, preparing food), plan and participate in outings and activities with family members, discuss and cooperate with plans according to other family members' needs.

Socio-Communication Skills: e.g., to be able to play/ interact with peers, ask for things and for help, answer questions, pass on messages, take turns during conversations and activities, express emotions.

Health & Safety: e.g., to be able to maintain hygiene, cross roads safely, take medications by self, know whom to share personal information with (like home address), avoid unsafe situations and people.

Executive Function Skills: e.g., to be able to develop skills like organization, planning, problem-solving, sequencing information, imagination & abstract thinking, regulating emotions.

Leisure/Recreation: e.g., to be able to spend time alone or with others watching a movie, reading, listening to music, playing a sport, going to a restaurant.

Motor Skills: e.g., to be able to tie shoelaces, button a shirt, cut with scissors, write, catch a ball, cycle, run with changes in direction.

Vocational Training: Job-specific technical training in computers, culinary skills, office skills,

gardening, etc. that will help with employment in the future.

**INTERVENTIONS**

Certain interventions provide support to individuals with ASD.

Please imagine that you had to choose from a list of INTERVENTIONS that will be most helpful for an individual with ASD. Based on your experience, please assign positions to each of these INTERVENTIONS by putting the most important at the top and the least important at the bottom.

You may use the up and down arrow, or drag and drop option to adjust positions.

Mental Health Counseling: Supports individuals through talking with trained professionals like psychologists etc.

Ayurveda: Involves medical interventions, dietary modifications, yoga and pranayams that

help in promoting physical and psychological health.

Speech and Language Therapy: Assists the individual in making clearer speech sounds, matching emotions with the correct facial expression, understanding body language, responding to questions.

Art, Music, Drama & Dance, Therapy: Engages individuals in these activities with systematic therapeutic guidelines and goals.

Pharmacological Treatment: Taking allopathic medications to help regulate emotion and mood, and manage other co-occurring conditions like epilepsy, through taking allopathic medicines.

Applied Behavior Analysis & Naturalistic Interventions: Use of behaviour principles and various techniques like Discrete Trial Training, Modeling, Reinforcement Systems etc. where behaviours are broken down into their simplest components and rewarded for good performance. Some e.g., of Naturalistic interventions using child-led techniques are ESDM, Floortime, Son-Rise.

Animal Assisted Therapy: Using trained animals to engage with individuals in various activities with systematic therapeutic guidelines and goals.

Yoga, Meditation, Sports and Fitness: Helps with physical and mental health through games like basketball, roller skating, table tennis, badminton, gym, swimming, Zumba, among others.

Occupational therapy: Includes physical activities to help develop coordination and body awareness, sensory integration, developmental activities such as brushing teeth and combing hair.

Nutritional Counseling: Provides individualized support for input regarding diet and

healthy eating habits.

**RESEARCH**

Below is a list of RESEARCH STATEMENTS that will be helpful for ASD.

Based on your experience, please assign positions to each of these RESEARCH STATEMENTS by putting the most important on top and the least important at the bottom.

*You may use the up and down arrow, or drag and drop option to adjust positions.*

I would like research to identify the most effective ways for the community to support people with ASD.

I would like research on how ASD can be diagnosed earlier.

I would like research on identification and interventions for co-occurring conditions with

ASD (e.g. lack of sleep, picky diet, anxiety, depression).

I would like research on how and why ASD changes over time.

I would like research on the wellbeing and safety of individuals with ASD.

I would like research on the causes of ASD.

I would like research on improving the way adults are diagnosed.

I would like research on interventions for ASD across the lifespan (including

pharmacological and behavioural interventions).

I would like research on gender and ASD.

**Supplementary Material 3: Interview questions**

| **Category** | **Questions** | **Conditions** | **Follow-up Questions** |
| --- | --- | --- | --- |
| ***General Information*** | Name of the child. |  |  |
|  | Which state do you belong to? |  |  |
|  | What is your/ your child’s age? |  |  |
|  | When did you/ your child receive a diagnosis?  What was the diagnosis given? |  |  |
|  | Who is the child’s primary caregiver? (Who spends the most amount of time with the child?) |  |  |
|  | How much time are you/ the caregiver able to spend with your child? |  |  |
| ***Skills*** | Why/ on what basis did you choose this order/ ranking? |  |  |
|  | How does this relate to your child? | (If not already answered through the previous question) |  |
|  | Would you like to change the ranking of the skills now? | If Yes: | How would you like to rank them now? |
|  | If your child were to choose, do you think he/ she will make the same choices? |  |  |
|  | Is there any skill that you think we have missed out on our list? |  |  |
| ***Intervention*** | Why/ on what basis did you choose this order/ ranking? | (If the answer is related  to accessibility, convenience and affordability, then ask: | Imagine all the interventions are made affordable, accessible, and available to you, what would be your top 3 preferences? |
|  | How does this relate to your child? | (If not already answered through  the previous question) |  |
|  | Are you satisfied with the range of services that are available to you today? |  |  |
|  | Would you like to change the ranking of the interventions now? | If Yes: | How would you like to rank them now? |
|  | If your child were to choose, do you think he/ she will make the same choices? |  |  |
|  | Is there any intervention that you think we have missed out on our list? |  |  |
|  | Any other therapies that the survey has missed out that you have personally tried for your child? |  |  |
| ***Research*** | Why/on what basis did you choose this order/ ranking? |  |  |
|  | Do you think your child would like to seek the same information? |  |  |
|  | How do you think research will help/be of use for autism? |  |  |
|  | How do you think we can best share the findings of autism research with the general population? | If they state that they don’t get to know  what research is happening: | Can suggest how they would like to read about them? |
|  | Would you have liked to change the ranking of the research statements now? | If Yes: | How would you like to rank them now? |
|  | Is there any research area that you think we have missed out on our list? | If Yes: | Please specify. |
| ***Source*** | From which sources do you get your information on autism? |  |  |
|  | Do you face any difficulty in understanding information available on autism? | If Yes: | Please specify. |
| ***The Survey*** | Were there any particular words/ statements/ sections in the survey that you found difficult to understand? |  |  |
|  | Do you know any other person who has autism (in or outside the family)? |  |  |
| ***Ending Question*** | Is there anything else that you wish to share or ask before we end? |  |  |

**Supplementary Material 4: Tables**

**Table 1 : Exploratory analysis: Descriptives**

| **Categories** | ***Options*** | ***Group (Income)*** | ***N*** | ***Mean*** | ***df*** | ***P value*** |
| --- | --- | --- | --- | --- | --- | --- |
| Skills  Research | Family support participation  Early Diagnosis | High  Low  High  Low | 89  181  89  181 | 5.01  4.34  3.35  2.28 | 268  268 | 0.043  0.028 |

Statistically significant differences in ranks were noted between respondents belonging to high vs low income groups in these two options within Skills and Research priorities. No other option showed a statistically significant difference in ranks between the two income groups.

**Table 2. Interviewee Demographics**

|  | | **n (%)** |
| --- | --- | --- |
| **Total** | | 40 (100) |
| **Gender** | |  |
| Male | | 27 (67.5) |
| Female | | 13 (32.5) |
|  | |  |
| **Age (in years)** | |  |
| < 7 | | 8 (20) |
| 8-13 | | 13 (32.5) |
| 19-25 | | 8 (20) |
| 26-40 | | 2 (5) |
| 40+ | | 0 |
|  | |  |
| **Relationship** | |  |
| Parents of autistic individuals | | 36 (90) |
| Autistic adults | | 4 (10) |
| **Diagnosis Age (in years)** | |  |
| < 7 | | 30 (75) |
| 8-13 | | 6 (15) |
| 19-25 | | 2 (5) |
| 26-40 | | 2 (5) |
| 40+ | | 0 |
|  | |  |
| **Income (in INR)** | |  |
| <50000 | | 7 (17.5) |
| 50000 -100000 | | 11 (27.5) |
| 100000-200000 | | 10 (25) |
| >200000 | | 12 (30) |
| **Support Needs** | |  |
| High support needs | | 14 (35) |
| Moderate support needs | | 17 (42.5) |
| Low support needs | | 9 (22.5) |
| **State of residence** | | |
| Known | | 6 (15) |
| Unknown | | 34 (85) |
| **Total time taken to complete the survey** |  |  |
| ≤5 minutes | | Excluded from the study |
| 5 mins to 29 mins | | 32 (80) |
| ≥ 30 minutes | | 8 (20) |
